# Evaluation of a knowledge transfer scheme to improve policy making and practices in health promotion and disease prevention setting in French regions: a realist study protocol

**DOI:** 10.1186/s13012-017-0612-x

**Published:** 2017-06-29

**Authors:** Linda Cambon, Audrey Petit, Valery Ridde, Christian Dagenais, Marion Porcherie, Jeanine Pommier, Chrisine Ferron, Laetitia Minary, François Alla

**Affiliations:** 1 UMR 6051 (CRAPE-Arenes), EHESP, Paris, France; 2 EA 4360, APEMAC, Université de Lorraine, Nancy, France; 3 Department of Social and Preventive Medicine, University of Montreal School of Public Health (ESPUM), Montreal, Canada; 4 University of Montreal Public Health Research Institute (IRSPUM), Montreal, Canada; 5 Department of Psychology, University of Montreal, Montreal, Canada; 6 Fédération Nationale d’Education et de promotion de la Santé (FNES), Paris, France; 7 EHESP, Paris, France

**Keywords:** Knowledge transfer, Realist evaluation, Complex intervention, Prevention, Public health

## Abstract

**Background:**

Evidence-based decision-making and practice are pivotal in public health. However, barriers do persist and they relate to evidence properties, organisations and contexts. To address these major knowledge transfer (KT) issues, we need to rethink how knowledge is produced and used, to enhance our understanding of decision-making processes, logics and mechanisms and to examine the ability of public health services to integrate research findings into their decisions and operations. This article presents a realist evaluation protocol to assess a KT scheme in prevention policy and practice at local level in France.

**Methods/design:**

This study is a comparative multiple case study, using a realist approach, to assess a KT scheme in regional health agencies (ARS) and regional non-profit organisations for health education and promotion (IREPS), by analysing the configurations contexts/mechanisms/outcomes of it. The KT scheme assessed is designed for the use of six reviews of systematic reviews concerning the following themes: nutrition, alcohol, tobacco smoking, physical activity, emotional and sexual life and psychosocial skills. It combines the following activities: supporting the access to and the adaptation of scientific and usable evidences; strengthening professionals’ skills to analyse, adopt and use the evidences in the course of their practices and their decision-making process; facilitating the use of evidence in the organisations and processes. RAMESE II reporting standards for realist evaluations was used.

**Discussion:**

The aims of this study are to experiment and characterise the factors related to the scheme’s ability to enable public health stakeholders to address the challenges of KT and to integrate scientific knowledge into policy and practice. We will use the realist approach in order to document the parameters of successful KT strategies in the specific contexts of preventive health services in France, while seeking to determine the transferability of such strategies.

## Background

Evidence-based decision-making and practice are major issues in public health. For researchers, this means looking ahead to the dissemination of findings and integrating different types of knowledge and decision-making challenges [1]. It also implies greater collaboration between the research community and decision-makers [2]. Public health research issues have to be approached alongside societal and health issues too. It follows that evidence-based policy-making and planning in public health offer a way to improve the efficiency, credibility, and sustainability of health systems [1]. Furthermore, this can lead to a better social acceptance of the chosen decisions and interventions [3].

Despite the general agreement about the interest of evidence informed practices and policy-making (EIDM), barriers do persist in both the production and use of evidence. These barriers relate to people, organisations, contexts and properties of evidences [4]. To address this, it is necessary to rethink how knowledge is produced and used, to enhance our understanding of decision-making processes, logics and mechanisms and to examine the ability of public health services to integrate research findings into their decisions and operations. This requires a systemic approach, which includes the adaptation of scientific knowledge, the ability of users to capture, understand and apply the available evidence, as well as an accurate organisation and a supportive culture for using evidence. These are the major challenges of KT, defined by the National Public Health Institute of Quebec (INSPQ) as “the group of activities and interaction mechanisms that foster the dissemination, adoption and appropriation of the most up-to-date knowledge possible for use in professional practice and in healthcare management” [5].

### What stands in the way of the use of scientific evidence in public health?

In France, there is no formal and structural KT scheme. There are a few initiatives led by the National Public Health Agency (ANSP) and the National Cancer Institute (INCA), which for instance produce literature reviews. But policy-makers and prevention professionals do not use them. It confirms that a passive diffusion of knowledge is not effective, and the effectiveness of KT strategies depends on the context in which they are implemented [6–10]. The contextualization of the KT strategies is necessary to remove barriers to knowledge use. According to Gervais et al. [3, 11], KT research on decision-making processes offers a number of explanatory factors which may be classified in three categories. The first relates to the specific properties of the evidence itself: nature, availability, accessibility, quality and credibility (data and sources), intelligibility, ability to meet needs, adaptability and transferability [3]. The second category relates to the personal characteristics of decision-makers: beliefs or personal values, political leanings, socio-demographics, level of education, previous experiences, motivation and ability to interpret data, etc. All of them may influence how new knowledge is addressed in the decision-making process.[12]. The third category refers to the characteristics of the organisations and local contexts in which knowledge producers and users work [4]: openness to change, material, human and financial resources available for KT, social and political context in the external environment, style of management, leadership, staffing, stakeholder coalitions, etc. Consequently, the multiple barriers to the adoption of evidence in the field of public health underline the non-linear process between knowledge production and knowledge use. If these barriers are to be overcome, we need to address all the parameters that affect the decision-making process. This is a focal point for KT research.

### The mechanisms of an effective knowledge transfer

Various strategies are available to overcome barriers to the use of KT. A recent work conducted by Langer et al. identified six mechanisms involved in effective KT:

- “Awareness” (M1) is defined as building awareness for, and positive attitudes toward, evidence-informed decision-making (EIDM). This mechanism emphasises the importance of decision-makers’ valuing the concept of EIDM.
- “Agree” (M2) is defined as the building mutual understanding and agreement on policy-relevant questions and the kind of evidence needed to answer them. This mechanism emphasises the importance of building mutual understanding and agreement on policy questions and what constitutes fit-for-purpose evidence.
- “Communication and access” is (M3) defined as providing communication of, and access to, evidence. This mechanism emphasises the importance of decision-makers receiving effective communication of evidence and convenient access to it.
- “Interact” (M4) is defined as the interaction between decision-makers and researchers. This mechanism emphasises the importance of decision-makers interacting with researchers in order to build trusted relationships based on mutual trust, collaborate, and gain exposure to a different type of social influence.
- “Skills” (M5) is defined as supporting decision-makers to develop skills in finding and making sense of evidence. This mechanism emphasises the importance of decision-makers’ having the necessary skills to identify, appraise, synthesise evidence, and integrate it with other information and political needs.
- “Structure and process” (M6) is defined as influencing decision-making structures and processes. This mechanism emphasises the importance of decision-makers’ psychological, social and environmental structures and processes (e.g. personal models, professional norms, habits, organisational and institutional rules) in providing means and barriers to action.

The authors underline that these strategies are effective if combined and contextualized in their implementation setting, confirming previous work of Ridde et al. [13] and Barwick [14]. Consequently, we hypothesize that in France, as elsewhere, simple diffusion and “one size fits all” strategies are not effective.

In this paper, we present the protocol of a realist evaluation study of knowledge transfer strategies implemented in the field of health prevention at a local level in France. We have used RAMESE II reporting standards for realist evaluations [15].

## Study objectives and location

The objective of the study is to identify the configurations contexts/mechanisms/outcomes of an effective KT scheme in local prevention sector. This study will be conducted in four French regions and within two types of organisation and their partners: regional health agencies (ARS), which are responsible for policy-making and prevention policies; and non-profit organisations (IREPS). IREPS develop health promotion and prevention programs and provide methodological supports to field professionals for the implementation of prevention interventions in different settings (work places, schools, care settings, recreation and community centres, rural or urban areas, etc.). ARS and IREPS work together to implement prevention and health policies in local contexts.

## Methods/design

We have reported this manuscript in line with the RAMESES II reporting standards for realist evaluation.

### Study design and conceptual framework

This study is a comparative multiple case study of a KT scheme in the field of health prevention using a realist approach [16, 17]. It concerns French public health services: ARS and IREPS. The case study design is the more suitable research strategy to investigate a phenomenon within its context and analyse this phenomenon’s interactions with several other elements relevant for our area of study [18].

The realist approach [17] is increasingly used for appraising the interactions between an intervention, its mechanisms and its contexts. The overall aim is to achieve a better understanding of an intervention’s success factors and how these may be replicated in other contexts. This type of evaluation examines what works, under what conditions and for whom, based on a middle-range theory (or configurational theory) which describes the interactions between outcomes, mechanisms and contexts [17, 19]. Thus, realist evaluation integrates the paradigm of black box evaluation [20]. While the experimental paradigm evaluates effectiveness without appraising an intervention’s mechanisms of impact, realist evaluation answers the following question: did the intervention work according to the theory underpinning it? This type of evaluation seeks to understand the intervention by focusing on its mechanisms and the influence of context. The mechanism is defined in this case as the “part of a participant’s response to an intervention, generally hidden and sensitive to variations in context, and which produces effects” [21]. In realist evaluation, causality is generative, meaning that what generates the effect relates specifically to the interactions between context and cause (here, the intervention methods) [19]. However, as we will study the patterns between these interactions in different contexts, we hypothesise that it is possible to isolate key elements that may apply across a set of contexts. These findings will thus generate intermediate theories that will be sharpened little by little as each case will be investigated.

**Fig. 1:**
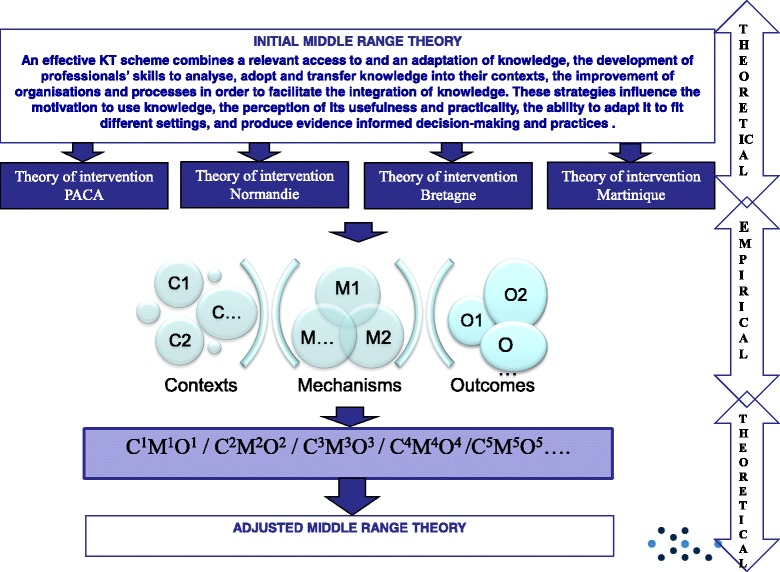
The realist sequences describes the empirical/theoretical sequences of the realist evaluation

To conduct a realist evaluation, we alternate theoretical and empirical stages (Cf. Fig. 1: The realist sequences). According to Langer’s work [2] and many authors [7, 10, 13, 14, 22], we hypothesize that an effective KT scheme has to combine an access to and an adaption of knowledge, the development of professionals’ skills to analyse, adopt and transfer knowledge into their contexts, the improvement of organisations and processes in order to facilitate the integration of knowledge. We also conducted an exploratory qualitative study in the four regions to collect data on the pre-existing scheme and activities related to KT and the potential local barriers. The questions were the following: what kind of KT activities are possible (types, timeline, duration, management)? Who may be involved? What structural/organisational mechanisms would be affected? What contextual factors, outside the control of those involved, would need to be addressed? Data will be collected by means of semi-structured interviews with IREPS directors and ARS public health directors (8 people). Based on the behaviour change wheel theory [23] and an exploration of the behavioural theories used in KT strategies [24], we hypothesize that the change of which will occur in knowledge use may be notably due to the motivation to use knowledge, the perception of its usefulness and practicality and the ability to adapt it in to fit different settings.

According to all the above scientific literature, and to support our realist evaluation, we built an initial middle range theory, defined as following: “An effective KT scheme combines a relevant access to and an adaptation of knowledge, the development of professionals’ skills to analyse, adopt and transfer knowledge into their contexts, the improvement of organisations and processes in order to facilitate the integration of knowledge. These strategies influence the motivation to use knowledge, the perception of its usefulness and practicality, the ability to adapt it to fit different settings, and produce evidence informed decision-making and practices”.

This initial middle range theory leads to the design of four theories of intervention one for each region describing the interventions, the expected outcomes, the contexts’ parameters and the expected mechanisms. This work will be conducted in a preliminary 2-day workshop, gathering ARS and IREPS professionals. These theories will be applied in the 4 regions, for 12 months, and data will be collected in order to characterize the contexts, mechanisms and outcomes and to determine the effective CMO configurations.

Finally, a cross-sectional analysis of the case studies will be conducted allowing us to identify potential regular CMO patterns, which would constitute an adjusted middle-range theory. The different stages are presented in Fig. 1 (Cf Fig. 1: The realist sequences).

### Intervention strategies

The intervention is a KT scheme designed for the use of policy briefs (PBs), which will be written on the basis of six reviews of systematic reviews (completed by international guidelines); an international scientific committee have carried out these reviews. They concern the following themes: nutrition, alcohol, tobacco smoking, physical activity, emotional and sexual life and psychosocial skills. These themes are primary in France. They present effective prevention practices.

Based on the report entitled “The science of using science: researching the use of research evidence in decision-making” [25], the scheme combines the following activities:

- Supporting the access to and the adaptation of scientific and usable evidences especially the policy briefs.
- Strengthening professionals’ skills to analyse, adopt and use the policy briefs in the course of their practices and their decision-making process (training, journal club, tutoring, etc.).
- Facilitating the use of evidence in the organisations and processes (collaborative workshops, normative processes, incentives, nudge, etc.).

According to the initial middle range theory and the 4 theories of change, professionals will make an action plan to apply them in their local settings. Theses KT activities will be set up over a 12-month period.

### Population

The targeted population is composed of prevention and public health services operating in French regions, namely, the ARS and IREPS and their partners. The study will focus on three groups of stakeholders:

- ARS public health professionals: five agents per region (deputy directors in charge of prevention, heads of strategy departments and project managers);
- IREPS professionals: ten people per region (directors, project managers and communication managers).
- Members of specialised prevention commissions within the Regional Conferences on Health and Autonomy (CRSA) and members of the Public Policy Coordination Commission (CCPP) both devoted to prevention in French regions (five people) and partners of IREPS and ARS.

We already have the agreement for the data collection given by the four ARS involved in the project since it began.

### Data collection

Data will be collected to document the support scheme’s mechanisms and contexts parameters involved in effectiveness. They will be collected before the implementation of the KT scheme at the end and throughout the implementation. They will be collected on the 3 categories of people described before; 20 people per each region (80 at all).

Collected data will characterise the context, the mechanisms relating to the organisation and to the individuals involved, the PBs and the set-up for KT.

A description of data collected and how and they will be collected are presented in Tables 1 and 2, but these variables will be adjusted according to the four theories of intervention and the action plans (Cf. Table 1 : Expected outcomes and Table 2 : Contexts and mechanisms expected).

**Table 1:** Expected outcomes

Stakeholders	Outcomes	Indicators	Data collection
ARS	Agents use policy briefs (PBs) in discussions at committee level	Number of verbatims per meetings Type of PBs or extracts from PBs Ways of using PBs	Semi-structured interview Observation
	Agents use evidences from PBs as criteria of project assessment	Existing in assessment grids	Documentary analysis Semi-structured interview
	Agents use evidences from PBs as part of conventional tools agreed between the ARS and its implementers (e.g. integration into specialised library and reference services)	Existence of mentioned PBs or extracts from PBs in documents Ways of using PBs	Documentary analysis Semi-structured interview
	Agents advocate evidences from PBs in their productions (communications, reports, action plans, etc.)	Number of communications, reports, action plans mentioning PBS or extracts from PBs	Semi-structured interview Observation Documentary analysis
IREPS	Professionals use evidences from PBs to design their projects	Number of projects mentioning PBs or extracts from PBs Ways of using PBs	Semi-structured interview Documentary analysis
	Professionals use evidences from PBs to evaluate their projects	Number of evaluation based on PBs or extracts from PBs Ways of using PBs	Semi-structured interview Documentary analysis
	Professionals use evidences from PBs to make reports to their sponsors	Number of reporting based on PBs or extracts from PBs Ways of using PBs	Semi-structured interview Documentary analysis
	Professionals use evidences from PBs in the methodological supports for field professionals	Number of methodological supports based on PBs or extracts from PBs Ways of using PBs	Semi-structured interview Observation
	Professionals advocate evidences from PBs in their productions (communications, reports, action plans, etc.)	Number of communications, reports, action plans mentioning PBS or extracts from PBs	Semi-structured interview Observation Documentary analysis
	Professionals use evidences from PBs as part of conventional tools agreed with their sponsors, included ARS and partners.	Existence of mentioned PBs or extracts from PBs in documents Ways of using PBs	Documentary analysis Semi-structured interview
Field professionals	Field professionals use evidences from PBs to design their projects	Number of projects mentioning PBs or extracts from PBs Ways of using PBs	Semi-structured interview Documentary analysis
	Field professionals use evidences from PBs to design their conventional tools with partners and sponsors	Existence of mentioned PBs or extracts from PBs in documents Ways of using PBs	Documentary analysis Semi-structured interview
CRSA	CRSA committee use evidences from PBs to make statements	Number of verbatim per meetings Type of PBs or extracts from PBs Ways of using PBs	Observation Documentary analysis Semi-structured interview
CCPP	CCPP committee use evidences from PBs to design their partnership aim, their common projects	Number of verbatim per meetings Type of PBs or extracts from PBs Ways of using PBs	Observation Documentary analysis Semi-structured interview

**Table 2:** Contexts and mechanisms expected

Types of variable	CMO	Types	Variables	Questions	Data collection
Context in each region (C)		Relating to regional policy-making and policy action on prevention	Leadership	Type of management Type of management structuring	Observation Documentary analysis Semi-structured interview
			How public health is organised	Funders Types of funding ways (competitive call for project, conventional agreement, etc.) Assessment of actions Main partnership between stakeholders.	Observation Documentary analysis Semi-structured interview
			Support mechanisms for stakeholders/practitioners	Types of supporting process Who support the practitioners Who are supported Who fund the supporting activities	Observation Documentary analysis Semi-structured interview
			Opportunities	Opportunities to work with researchers, to use evidences from researchers in practices	Observation Documentary analysis Semi-structured interview
			Collaborative	Experiences of collaborating work with researchers Assessment of them	Observation Documentary analysis Semi-structured interview
			Specific decision-making and operational process	Description of decision-making process Description of designing, setting and assessment of interventions	Observation Documentary analysis Semi-structured interview
Parameters influencing the use of the PBs	Mechanisms (M)	Relating to the PBs	Acceptability of PBs Closeness between practices and PBs recommendations Convenience of PBs with context and practices Credibility perceived of PBs Other mechanisms not expected		Observation Documentary analysis Semi-structured interview
		Relating to stakeholders/professionals	Ability to integrate new practices in the context, in the habits (capabilities) Interest from PBS using Culture of change existing (previous experiences, awareness, agreement) Motivation of using PBs Levels of interaction between researchers and practitioners to discuss about evidence-informed practices Other mechanisms not expected		Observation Documentary analysis Semi-structured interview
		Relating to organisations	Changes in ability to evolve (opportunities in functioning, hierarchical agreement, etc.) Temporality (opportunity to take time to introduce new knowledge coming from PBs) Other blocking or driving mechanisms not expected		Observation Documentary analysis Semi-structured interview
		Others	Other mechanisms not envisaged initially		Observation Documentary analysis Semi-structured interview
Conduct of the KT	Intervention (I) set up locally		Type of KT activity set up locally Duration of these activities (action plans) Types of activity carried out Stage of completion of the expected activities Contributors involved in KT strategies Partnerships involved in KT strategies Financial resources in KT strategies Material resources in KT strategies		Observation Documentary analysis Semi-structured interview

Data will be collected by means of:

- Semi-structured interviews conducted with the above-mentioned population (20 people per region)
- The observation of health promotion meetings and collective events resulting from the scheme’s implementation: project selection committees, selection processes, trainings, seminars, presentations etc. The aim of these observations is to study the types of interactions between the professionals who deal with KT strategies (for instance, leadership, uptake, bottom up or top down approaches).
- A documentary analysis (calls for project, action plans, projects applications, reports of meeting, etc.)

The observation and documentary analysis grids and the interview guideline will be designed based on the four intervention theories and tested on a sample of five stakeholders not involved in the process, but belonging to the IREPS network.

The collection will last 12 months.

### Data analysis

Data will be processed through a content analysis [26] defined as “A set of systematic and objective procedures for analysing communication processes in order to obtain indicators (quantitative or not) inferring knowledge related to the conditions (inferred variables) under which meaningful information is both sent and received”. This analysis will code, classify and grade content in order to identify patterns, trends and specific features. We will use a software program called *Nvivo* to assist us in conducting and integrating a thematic analysis of the interviews and an analysis of the observation reports. The qualitative analysis will lead to:

- Document the uptake of evidence and the practice changes triggered by the intervention. This will be carried out on a case-per-case basis in monographic format, in order to identify the mechanisms at play, the degree of intervention, the contextual contingencies and the changes arising in the three types of knowledge use (instrumental, conceptual, persuasive).
- Identify the most regular CMO configurations by a cross-analysis of the different cases and a combination of the different data collected according to their linkage with the “context” meanings, “mechanism” meanings and “outcomes” meanings (cf Tables 1 and 2).

Based on the observed elements, we will classify the outcomes in three categories of use as recorded in the literature [27, 28].

- Instrumental use: knowledge users draw on the IBs to make decisions or to change their practice;
- Conceptual use which implies changes in understanding and thinking inspired by the IBs;
- Persuasive use (also called strategic or symbolic use) which refers to a use of knowledge as a means to justify decisions or actions.

We will process these data to characterise the mechanisms identified in the intervention theory and induced by the intervention. This will enable us to answer the following questions about several mechanisms related to knowledge, people and organisations: (1) were they present in the contexts studied? (2) Did they positively or negatively influence the outcomes from a user’s perspective? (3) Which mechanism(s) was (were) actually active (which parameter influenced which other parameter and/or which outcome)? (4)Which outcome(s) was (were) produced?

Then, a secondary middle range theory will then be developed, leading to future guidelines.

### Development of an adjusted middle range theory

Based on this analysis, we will compare the CMO configurations, which will be identify with the initial middle range theory, and we will figure out an adjusted middle range theory. This work will be conducted during a second interdisciplinary workshop, based on a discussion about analysis from data collected, gathering all ARS staff responsible for prevention and public health, IREPS directors and project officers, plus agents from ANSP and INCA.

KT development guidelines in France will emerge from this meeting.

### Communication and dissemination of results

Different types of actors will be involved throughout the study: prevention professionals, policy-makers and researchers. Thus, multiple methods will be used to communicate research results:

- Developing short and practical policy briefs about knowledge transfer to national policy-makers and practitioners
- Delivering presentations at local, national meetings in France and relevant international meetings for professionals and researchers
- Regular project review meetings and continuous engagement with key decision-makers and practitioners, in particular as part of the Public Health Initiative for the Interaction between Research, Intervention and Decision-*Initiative en Santé Publique pour l’Interaction entre la Recherche, l’Intervention et la Décision* (InSPIRe-ID), a knowledge transfer consortium, led by the French Ministry of Health.
- Delivering presentations at national and international conferences and publishing articles in peer-reviewed academic journals with emphasis on open access
- Developing a project research report for the funder, with a publishable executive summary

## Discussion

This article describes a protocol using a realist design to understand how a KT scheme works, for whom and in what circumstances. In research, realist evaluation is valuable for evaluating interventions in their contexts; it addresses contextual factors in relation to the mechanisms and outcomes of these interventions. Thus, partial patterns can be revealed to explain how interventions may foster enhanced KT.

However, even if there is weak support in France to develop KT at a local level, KT is highly recommended by health national authorities. Consequently, we need to address a potential social desirability bias, resulting both from the subject and the fact that the data are not self-reported [28]. This bias results from the tendency of survey respondents to answer questions in a manner that will be viewed favorably by others. Moreover, we will design the interventional scheme with the different stakeholders. Researchers are thus involved in the assessed process. This contextual parameter must be taken into account in the evaluation.

The aim of this study is to experiment and characterise the success factors of a KT scheme in health promotion and disease prevention settings. By success, we mean the scheme’s ability to (1) enable public health stakeholders to address the challenges of KT and (2) bring about changes in public health policy and practice: integration of evidence-informed public health, collaborative practices etc. We will seek to explain the parameters and conditions of these strategies in order to determine their transferability into other contexts. This will provide a basis for the production of operational and contextualised guidelines in order to develop KT to inform regional policy-making on health promotion and disease prevention. Ultimately, this research aims at enhancing overall policy-making and quality of implementation in the sector. With this in mind, this project will be of great interest for public policy-makers who are currently moving towards evidence-informed health promotion and disease prevention in France.

## Abbreviations

ANSP: Agence Nationale de Santé Publique (National Agency for Public Health)
ARS: Agence Régionale de Santé (Regional Health Agency)
CCPP: Commission de Coordination des Politiques Publiques (Public Policy Coordination Committee)
CRSA: Conférence Régionale de la Santé et de l’Autonomie (Regional Conference on Health and Autonomy)
FNES: Fédération Nationale d’Education et de promotion de la Santé (National Federation for Health Education and Promotion)
I: Intervention
IB: Intervention brief
INCa: Institut National du Cancer (National Cancer Institute)
InSPIRe-ID: Initiative en Santé Publique Pour l’Interaction entre la Recherche, l’Intervention et la Décision (a public health initiative dealing with the interaction between research, intervention and decision-making)
INSPQ: Institut National de santé publique du Québec (Quebec Public Health Expertise and Reference Centre)
IREPS: Instance Régionale d’Education et de Promotion de la Santé (a non-profit organisation promoting health at a regional level)
KT: Knowledge translation
PACA: Provence-Alpes-Côte-d’Azur (region in the south of France)
